# Excess water storage induced by viscous strain localization during high-pressure shear experiment

**DOI:** 10.1038/s41598-019-40020-y

**Published:** 2019-03-05

**Authors:** Jacques Précigout, Holger Stünitz, Johan Villeneuve

**Affiliations:** 1 Institut des Sciences de la Terre d’Orléans (ISTO), UMR 7327, CNRS-BRGM, Université d’Orléans, Orléans, France; 2 Department of Geology, University of Tromsø, Tromsø, Norway; 3 Centre de Recherches Pétrographiques et Géochimiques (CRPG), Nancy Université, CNRS, Vandoeuvre-lès-Nancy, France

## Abstract

Strain localization in viscously deformed rocks commonly results in fine-grained shear zones where massive fluid circulation is regularly observed. Recently attributed to strain-induced pumping, this phenomenon may have major implications for the distribution of ores deposits and rock rheology. However, although grain size reduction and/or creep cavitation have been proposed as important processes, the source mechanism of fluid concentration remains unresolved, particularly at high pressure. Here we use secondary ion mass spectrometry to document the H_2_O content of fine-grained olivine across an experimental shear zone, which developed with grain size reduction during a H_2_O-saturated shear experiment at 1.2 GPa and 900 °C. Through data interpolation, the olivine matrix reveals high fluid concentrations where shear strain is localized. These concentrations far exceed the predicted amount of H_2_O that grain boundaries can contain, excluding grain size reduction as a unique source of water storage. Instead, we show that H_2_O increases per unit of grain boundary across the shear zone, suggesting that cavitation and “healing” processes compete with each other to produce a larger pore volume with increasing strain rate. This provides an alternative process for fluids to be collected where strain rate is the highest in deep shear zones.

## Introduction

On Earth, both the crust and mantle incorporate aqueous fluids that interact with solid rock materials in many ways. Commonly referred to as fluid-rock interactions, they strongly affect rock deformation and petrogenetic processes, giving rise, for instance, to hydrolytic weakening, pressure-solution creep or metamorphic reactions, including partial melting. During strain localization, this type of interactions may involve one or several chemo-physical processes that help to channelize fluid flow in ductile shear zones where grain size is substantially reduced. When rocks deform by sub-solidus viscous creep, strain indeed partitions into fine-grained shear bands that recurrently develop in the presence of massive fluid circulation, as revealed by the enrichment of hydrous phases^1–4^ in the shear zones. Although the source process of such fluid flow localization is unknown at present, it may have critical implications for rock mechanics and distribution of ore deposits in deep Earth environments^5^.

Primarily attributed to seismic pumping of a pre-existing fault^1^, fluid infiltration is commonly inferred using evidence for dissolution-precipitation, which suggests a long-term process rather than a co-seismic one^3,5–7^. Rutter and Brodie^8^ and Wark and Watson^9^ proposed that high fluid contents could occur in ductile shear zones as a result of fluid permeation in response to grain size reduction; because of high pressure and high temperature, supercritical fluids may distribute along grain boundaries as a uniform boundary film^9^. However, recent observations of syn-tectonic water accumulation along mantle shear bands^4^ do not support such a “passive” process for attracting fluids towards ductile shear zones. Instead, they suggest that fluids are dynamically driven by the deformation itself.

There is abundant documentation of micro-pores produced during deformation of fine-grained material, including within natural shear zones in the middle/lower crust^5,7,10–15^ and during deformation experiments on ceramics, metals and natural rocks^16–20^. Also referred to as creep cavitation, the production of these micro-pores results either from grain boundary sliding (GBS) or, in a minor extent, from Zener-Stroh cracking if dislocations interact with grain boundaries^17^. In both cases, the micro-cavities arise from limitations of the material to flow, particularly when diffusive mass transfer is slow at low temperature. This led several authors to propose that strain-induced cavities in fine-grained shear zones may fill with fluids during deformation, constituting a potential alternative for surrounding fluids to be pumped^5,7,14^.

However, evidence of fluid pumping induced by creep cavitation is rather circumstantial. As direct observation of fluid infiltration is still lacking, such interaction only relies on the coexistence of micro-pores and tracks of fluid circulation in some crustal shear zones^5,15^. Furthermore, as creep cavitation is a process producing pressure-dependent dilatancy^21^, could we expect cavitation to occur if lithostatic pressure increases and limits dilatancy? The role of creep cavitation in fluid infiltration within ductile shear zones therefore remains uncertain, particularly at high pressure.

In this study, we used Secondary Ion Mass Spectrometry (SIMS) and Electron Backscatter Diffraction (EBSD) analyses to determine the respective distributions of H_2_O content and grain size across a shear zone produced experimentally at 1.2 GPa and 900 °C. This deformation experiment highlights strain-related fluid concentration that arises with an increase of H_2_O per unit of grain boundary. Providing the first experimental evidence of excess water storage in a ductile shear zone, our dataset is here described and discussed in the view of current hypotheses.

## Results

### Deformation experiment and SIMS analyses

**Figure 1:**
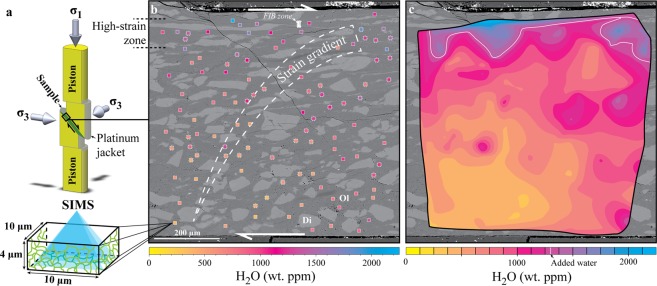
Distribution of H_2_O across the experimental shear zone. (**a**) Three-dimensional section of the sample assembly used to hot-press and deform 70 wt% of fine-grained olivine (~2 µm) plus 30 wt% of coarse-grained diopside (~100 µm) at 1.2 GPa and 900 °C. While the pressure (σ_3_) is applied using NaCl as the confining medium, the sample is deformed within a platinum jacket by pushing on alumina pistons (σ_1_) partly cut at 45° of the piston axis^25^ (non-coaxial assembly; see Supplementary Fig. S1). After deformation, the H_2_O content is documented for the olivine matrix using secondary ion mass spectrometry (SIMS) in spots of 10 × 10 × 4 µm^3^, which necessarily includes several grain boundaries (green lines). (**b**) Backscattered electron (BSE) image of the strain gradient that results from localization of deformation in the top part of the sample (high-strain zone). Each spot and related colour coding refers to the measured H_2_O content, including the crystal lattice and grain boundaries. All analyses surrounded by a dotted circle are used in Fig. 3. (**c**) Mapping of the SIMS dataset across the strain gradient. The interpolation was processed using the *griddata* function of MATLAB with isocontours increment of 125 ppm. The white line gives the isocontour of added water before experiment. Ol = olivine; Di = diopside; FIB = Focused Ion Beam zone documented in Précigout and Stünitz^25^.

The experiment was performed in a Tullis-modified Griggs-type apparatus on ~2 µm grain size San Carlos olivine and ~100 µm grain size Cranberry lake diopside sorted and mixed at a ratio of 70:30 wt%, respectively. Following a 24-hours period of hot-pressing, the powder was deformed at 900 °C and 1.2 GPa in a non-coaxial solid-salt sample assembly, which includes a platinum jacket welded at both ends around the sample^22–24^ (Fig. 1a). We added 0.2 μl of distilled water before welding the jacket, corresponding to ~1330 wt. ppm with respect to the sample powder. The olivine grain size has been chosen in a suitable range for diffusion creep deformation at an applied strain rate of ~2.10^−5^ s^−1^, giving rise to peak and steady-state flow stresses at ~0.95 and ~0.7 GPa, respectively (see Supplementary Fig. S1). For further details on the deformation experiment and related microstructures, the readers are referred to Précigout and Stünitz^25^.

In the sample, strain localized within a narrow zone of ~50 µm width where the olivine grain size is reduced from ~2 µm to ~1 µm equivalent diameter. The resulting strain gradient is highlighted by the progressive alignment and increasing aspect ratio of diopside grains towards the high-strain zone (Fig. 1b), where shear strain can reach more than gamma = 10^25^. Using SIMS, we measured the OH, silica and oxygen contents of the olivine matrix to quantify the amount of H_2_O across the strain gradient (Fig. 1b and methods). Considering the olivine grain size and ionized volume of a SIMS pit (10 × 10 × 4 µm^3^; see Supplementary Fig. S2), each analysis includes H_2_O from both the grain boundaries and olivine crystal structure (Fig. 1a). The analyses also include the presence of fluid in micro-cavities, as revealed by transmission electron microscopy performed on a focused ion beam section in the high-strain zone^25^ (Fig. 1b).

### Distributions of H_2_O and grain size

In Fig. 1c, we have mapped our SIMS data to show the distribution of H_2_O (see methods). The data interpolation indicates ~1500 ppm and locally more than 2000 ppm where strain is highest. These values are far above the 1330 ppm of bulk water added before the experiment, particularly if we consider the loss of hydrogen during hot-pressing and deformation, here estimated at ~550 ppm H_2_O from a hot-pressed, undeformed sample (see Supplementary Fig. S1). In contrast, the olivine matrix contains about 400 ppm of H_2_O where strain is lowest, giving rise to a gradient of fluid content correlated with the strain gradient. Although some local spots of moderate to high H_2_O content are observed outside of the high-strain zone, the first-order distribution of H_2_O content is not correlated with the distribution of macro-porosity, which remains constant (see Supplementary Fig. S3). Each value also exceeds the amount of H_2_O the olivine lattice can incorporate in these experimental conditions, i.e., ~150 ppm^26^.

**Figure 2:**
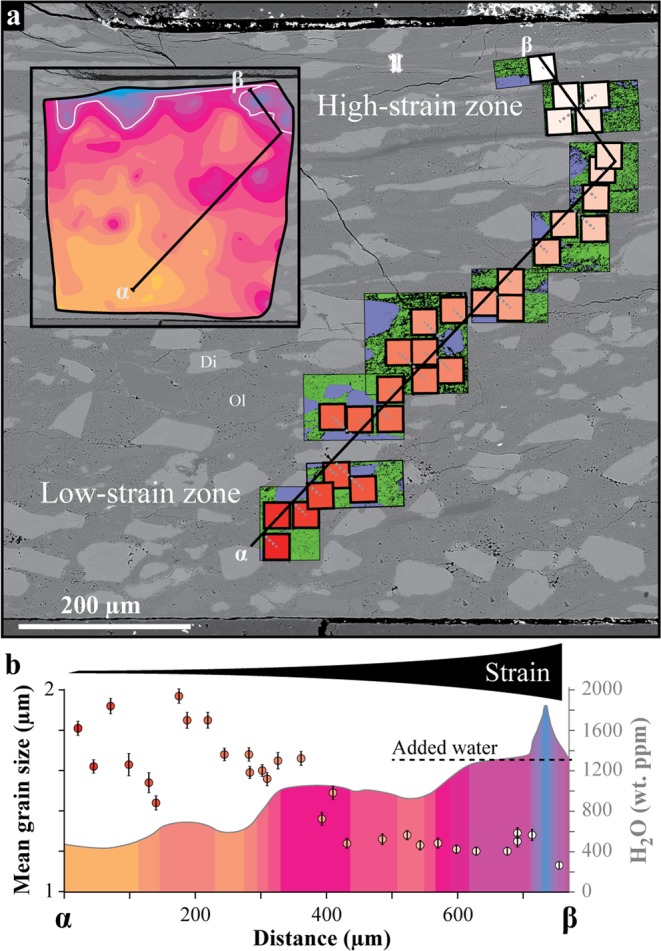
Grain size vs. H_2_O content. (**a**) BSE image of Fig. 1b and electron backscatter diffraction (EBSD) maps used to calculate the olivine grain size from α to β. While olivine and diopside are respectively shown in green and purple with grain boundaries (black thin lines), the mean grain size has been calculated on 30 × 30 µm^2^ areas through all of these maps (also available in Supplementary Fig. S5). Each value of grain size has been positioned so that their respective area centre was orthogonally projected to the cross-section line (grey dotted lines). The colour coding refers to the position of each value along the cross-section, from red in the low-strain zone (α) to white in the high-strain one (β). The pores, cracks, and non-indexed points are shown in black on EBSD maps. (**b**) Cross-section of H_2_O content combined with grain size evolution from α to β (the location of the cross-section with respect to H_2_O distribution is shown in the top left inset of (**a**). The range of colour from red to white refers to the 30 × 30 µm^2^ areas shown on (**a**). Each value of mean grain size is given with error bar (vertical solid lines), such as calculated using the standard deviation of their respective grain size distribution (see Supplementary Table S2). With increasing strain, the grain size and H_2_O content show an opposite and partially correlated behaviour; while grain size homogenizes and decreases, the H_2_O content increases and exceeds the amount of added water in the high-strain zone. Ol = olivine; Di = diopside.

Based on electron backscatter diffraction (EBSD) maps, we further document the olivine grain size across the strain gradient. Along a cross-section that extends from the low-strain (α) to high-strain (β) zones, we used 30 maps of ~30 × 30 µm^2^ with a resolution of 0.1–0.2 µm step size to compare the mean grain size and amount of H_2_O (Fig. 2a). From α to β, the grain size is heterogeneous and varies by ± 0.3 µm around an equivalent diameter of 1.7 µm. Such heterogeneity also characterizes the undeformed reference sample that has been hot-pressed at the same conditions of pressure, temperature and duration (67 h; see Supplementary Fig. S4). By approaching the high-strain zone after a 300 µm wide zone of low strain, the grain size becomes more uniform and progressively reduces to 1.15 ± 0.1 µm in the high-strain zone (Fig. 2b). The minimum grain size is observed in the high-strain zone where the H_2_O content exceeds the initial amount of water added. This correlates well with the H_2_O content that increases with reducing grain size, but only above 800 ppm. While the amount of H_2_O progressively increases along the cross-section, the grain size does not correlate with the H_2_O content below 800 ppm, i.e., at more than 400 µm from the high-strain zone. Thus, grain size and H_2_O content are only partially correlated.

### Evidence of excess water storage

**Figure 3:**
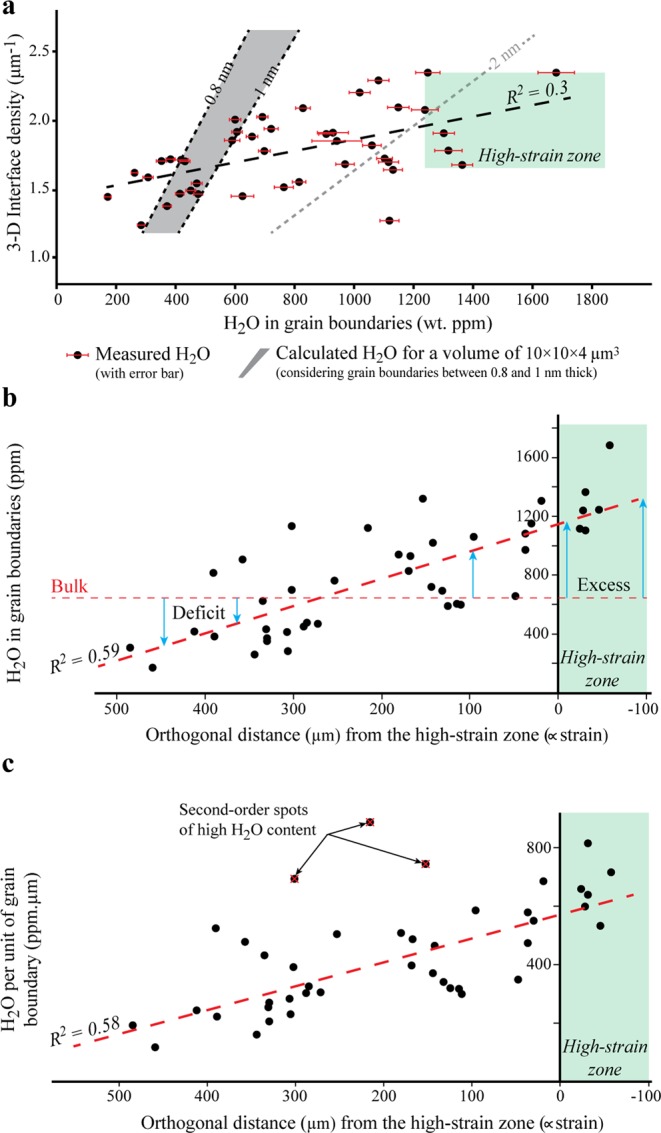
Evidence of excess water storage in grain boundaries. (**a**) Interface density versus H_2_O content in grain boundaries, such as estimated from the measured amount of H_2_O (black points) with error bar (red solid lines) minus the 150 ppm stored in the olivine crystal structure^26^. Using 42 SIMS analyses (located in Fig. 1b), the linear best-fit correlation of our data points (dotted black line) indicates a coefficient (R^2^) of 0.3. For comparison, we give calculations of the maximum amount of H_2_O that grain boundaries can contain within a volume of 10 × 10 × 4 µm^3^, and considering boundaries between 0.8 and 1 nm thick (grey area). An estimation is also given for a boundary thickness of 2 nm (dotted grey line). These estimations are based on grain boundary densities calculated on 20 × 20 µm^2^ EBSD maps (see Supplementary Fig. S5), and then extrapolated in three dimensions (3-D) through multiplication by 4/π^27^. The green dotted rectangle shows the range of density and H_2_O content documented in the high-strain zone. (**b)** H_2_O content in grain boundaries versus strain, here depicted as the orthogonal distance from the high-strain zone. In this case, the best-fit correlation (dotted line) indicates a R^2^ of 0.59. We also give the bulk H_2_O content, which corresponds to the amount of water added before experiment (1330 wt. ppm) minus the loss of H_2_O during the experiment (550 wt. ppm deduced from a hot-pressing sample; see Supplementary Fig. S1) and H_2_O stored in crystals structure (150 wt. ppm)^26^. This highlights a deficit of water in the low-strain zone and excess in the high-strain one (see text). (**c**) Strain (i.e., orthogonal distance from the high-strain zone) versus H_2_O content per unit of grain boundary, as estimated through normalization of the values in (**a**) by their respective 3-D interface density. The best-fit correlation (dotted line) indicates that H_2_O increases with strain with a coefficient R^2^ of 0.58. This coefficient excludes a few anomalies (3) located far above the first-order trend.

In Fig. 3a, we plot our SIMS dataset with respect to the interface density in three dimensions (3-D). While the grain boundary areas have been estimated from the density of grain boundary length on EBSD maps multiplied by 4/π^27^ (see Supplementary Fig. S5), each value of H_2_O content is shown for grain boundaries only, i.e., the measured amount minus the potential one stored in the olivine structure (~150 ppm)^26^. For comparison, we also estimate the amount of fluid that grain boundaries can contain for a given interface density and grain boundary thickness (Fig. 3a). Based on previous studies that documented grain boundary thicknesses lower than 1 nanometer at pressures higher than 1 GPa^20,28^, we used a thickness of 0.8 to 1 nanometre within a volume of 10 × 10 × 4 µm^3^, which corresponds to the ionized volume of a SIMS analysis. These calculated values do not consider any additional volume connected or not to grain boundaries, such as strain-induced cavities or fluid inclusions.

Our calculations show that H_2_O content increases with increasing interface density according to a linear relationship. For instance, if grain boundaries have a thickness of 1 nm, we expect between 420 and 750 wt. ppm for the range of 3-D interface density documented in our sample, i.e., between 1.2 and 2.4 µm^−1^ (Fig. 3a). In contrast, the linear best-fit correlation of our SIMS dataset follows a very different trend characterized by lower H_2_O contents below ~1.6 µm^−1^, i.e., in the low-strain zone, and an excess of H_2_O above ~1.7 µm^−1^, i.e., close by or within the high-strain zone (Fig. 3a). Furthermore, the linear correlation of our dataset indicates a coefficient (R^2^) of 0.3, whereas a graph of the same dataset for H_2_O with respect to the shortest distance from the high-strain zone yields a positive correlation with a linear R^2^ of 0.59 (Fig. 3b). The distribution of H_2_O in grain boundaries thus correlates better with strain than with interface density. With respect to the bulk H_2_O content in grain boundaries, which corresponds to 1330 ppm (added H_2_O before experiment) minus 700 ppm (550 ppm potentially lost during experiment, plus 150 ppm stored in olivine), we confirm that grain boundaries are characterized by a deficit and then an excess of H_2_O with decreasing distance from the high-strain zone (Fig. 3b).

In Fig. 3c, through normalization of the amount of H_2_O by 3-D interface densities, we further provide an estimate of the H_2_O content per unit of grain boundary segment across the shear zone. This graph shows that H_2_O increases with decreasing distance from the high-strain zone according to two different signals: one that includes most of the data (39) with a good and positive linear correlation (R^2^ = 0.58) and a second one with a few data (3) out of trend and far above the linear correlation. Together with an excess water storage in the high-strain zone, our dataset therefore highlights 1) an increase of H_2_O per unit of grain boundary with increasing strain, and 2) a few local anomalies of high fluid content with respect to the first-order trend.

## Discussion

Because of the high confining pressure during the experiment, some pores have been produced while the sample was quenched and decompressed. These pores may still contain some fluid if they were not connected to the sample surface during SIMS analysis, and hence, documenting a few anomalies of H_2_O in excess with respect to the volume deduced from the interface density may be expected. However, a continuous increase with strain as described in Fig. 3 is not consistent with the occurrence of inhomogeneities induced by unloading.

In previous studies^8,9^, excess water storage in ductile shear zones was attributed to grain size reduction leading to fluid permeation into new grain boundaries. Although grain size reduction is documented here, the grain size is not correlated with the full distribution of H_2_O content across the shear zone (Fig. 2b). The calculated trends in Fig. 3a also do not align with our dataset correlation, even if we consider a boundary thickness of 2 nm, which would be needed to account for the surplus of H_2_O. This value is more than twice the thickness observed at pressures higher than 1 GPa^20,28^. Being well in excess of the error bars, these features thus do not support the inference of grain boundaries as unique storage sites for H_2_O. We therefore envisage two other possibilities that we discuss below: (1) an overall expansion of grain boundaries with increasing strain, so that more H_2_O can be included per unit of grain boundary; or (2) creep cavitation forming a volume fraction of cavities in excess of the interface density.

The occurrence of a non-uniform boundary film along grain boundaries is very unlikely to occur considering viscous flow at high confining pressure^9^. A thickness of more than 2 nm in the high-strain zone does not either match available data from high-pressure experiments^20,28^ (greater nominal thicknesses are observed only in shear bands with porosity on the micro-scale). In contrast, many experiments on metals indicate that creep cavitation is a direct function of strain rate, and hence, a function of strain acquired during a given period of time^17^. Indeed, uniaxial tensile experiments on aluminium, copper and steel have been performed at various temperatures that involve viscous strain^29–32^. They all describe a time-to-rupture that decreases with increasing strain rate, giving rise to the so-called Monkman-Grant relationship^33^. This suggests that the creep rate primarily controls both the nucleation rate and subsequent growth of cavities, such as required for the material to fracture through void coalescence^17,30,32^.

In nature, the production of strain-induced micro-cavities is depicted as a non-cumulative, transient phenomenon related to steady-state grain boundary sliding^5,7,14^. Thus, the occurrence of ductile failure in metals, which typically describes a non-steady-state flow, differs from natural samples where the rupture of the material is prevented by closing of the micro-pores. This requires the occurrence of “healing” processes that may include grain boundary migration and/or phase nucleation^4,5,7,34^, both of which depend on fluid-assisted temperature-dependent diffusional processes. The confining pressure may also help to suppress failure by cavitation. In our experiment, deformation occurred at high confining pressure and at stabilized conditions of flow stress and temperature^25^. The olivine matrix deformed by grain-size-sensitive creep with evidence of phase nucleation and grain boundary sliding, which were both active across the shear zone. Together with observations of strain-induced micro-cavities in the high-strain zone^25^, these features support creep cavitation to occur coevally with healing processes during steady-state flow. Considering our SIMS dataset as a proxy of the volume fraction of cavities, we therefore attribute fluid concentration to creep cavitation, either related to a production of more cavities in the high-strain zone, or owing to larger pore sizes as the strain rate increases.

**Figure 4:**
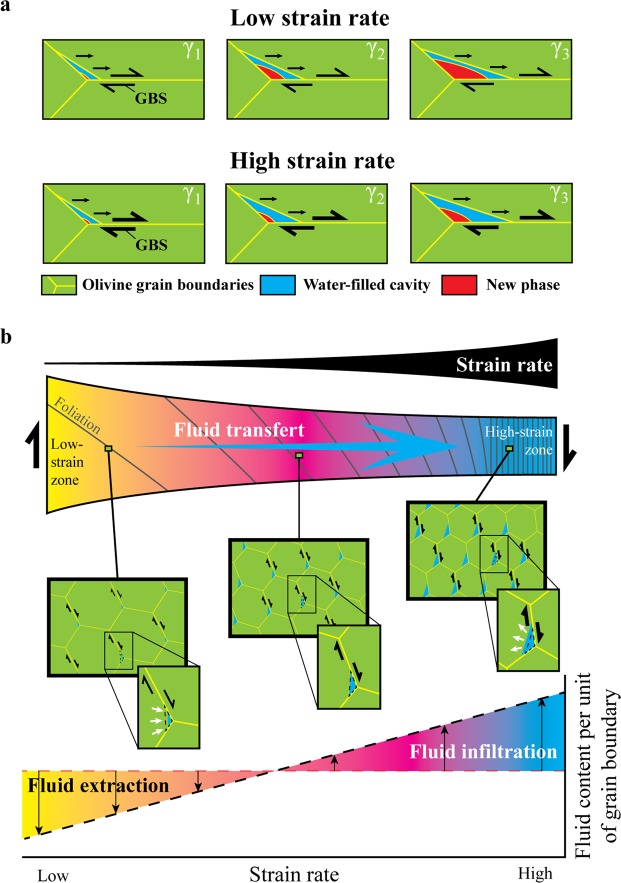
Model of fluid transfer induced by differential creep cavitation. (**a**) Changing size of strain-induced micro-cavities as a function of strain rate. Depending on the balance between strain rate and efficiency of “healing” processes, here represented by the nucleation and growth of a new phase, grain boundary sliding (GBS) is proposed to produce transient cavities with different sizes. While small water-filled cavities occur at low strain rate, larger pores are expected at high strain rate because of constant pressure and temperature that limit the growth rate of new phases in closing cavities. γ_1_, γ_2_ and γ_3_ represent three steps of finite shear strain. (**b**) Fluid transfer induced by differential creep cavitation across a ductile shear zone. Because of increasing strain rate, creep cavitation produces pores that gradually enlarge from the low- to high-strain zones. More cavities may be also expected for a higher contribution of GBS to deformation because of grain size reduction (not shown here). Fluids are consequently drawn and pumped towards the core of the shear zone until steady-state strain localization is achieved. The fluid gradient is then preserved as long as differential creep cavitation is effective, giving rise to a gradient of fluid content per unit of grain boundary with increasing strain rate (graph). The colour coding mimics the H_2_O distribution shown in Fig. 1 from yellow to light blue. Ol = olivine.

Although poorly quantified, the activity of GBS is expected to be inversely related to grain size during diffusion creep^35^: the smaller is the grain size, the higher is the contribution of GBS to deformation with respect to other deformation processes. Hence, a higher production of pores may be expected if the contribution of GBS increases with decreasing grain size. Nevertheless, our distribution of H_2_O content is better correlated with strain (or strain rate) than with grain size (Fig. 3), suggesting that the volume fraction of cavities is not only related to the cavitation rate. We thus hypothesize a relationship where excess water storage results from the production of pores with different sizes. In this model, here referred to as differential creep cavitation, the transient size of cavitation volume depends on the balance between cavity formation at a given strain rate and competing healing processes, which both control the opening and closing rates of pores (Fig. 4a). While a small (or absent) cavity volume is predicted at low strain rate because diffusional processes are fast enough to accommodate shape changes during grain boundary sliding, larger cavities are expected to occur at faster strain rates owing to the insufficient closure rate of cavities by diffusion.

Differential creep cavitation accounts for an initial production of cavities before strain localization, the size of which is the same. When strain is then partitioning and forming the shear zone, new micro-cavities are produced with different sizes depending on strain rate. While larger pores arise in the high-strain zone, smaller cavities are produced in the low-strain one (Fig. 4b). A higher cavitation rate may be also expected if the contribution of GBS to deformation increases with grain size reduction. As a consequence, H_2_O is progressively extracted and infiltrated through grain boundaries towards the high-strain zone until steady-state strain localization is achieved. At this stage, fluid transfer might no longer be effective across the shear zone, but transient micro-cavities are still produced with different sizes and/or at different rates, and the fluid migrates locally from closing to opening pores. The different size and/or number of micro-cavities is finally preserved *post-mortem* through quenching, giving rise to an apparent difference of H_2_O content per unit length of grain boundary (Fig. 4b).

This study therefore provides direct evidence for fluid concentration related to high-pressure viscous strain localization, highlighting ductile shear zones as able to channelize fluid flow in the deep Earth, and not only at upper levels of the lithosphere. As suggested in previous studies^4,5^, we also confirm that fluid permeation due to grain size reduction is not sufficient to account for this feature. Our findings suggest instead that fluids concentrate as a function of strain/strain rate and/or cavitation rate, here attributed to differential creep cavitation that produces water-filled cavities with different pore sizes and/or at different rates. While transient strain-induced cavitation increases and accommodate fluids where the strain rate increases, cavities are reduced and expel fluids from areas where the strain rate decreases. This may combine with an increasing cavitation rate while grain size is reduced. In this way, fluids are dynamically transferred towards regions of high strain rate and small grain size, as encountered in the core of deep shear zones.

## Methods

### Sample preparation

The deformation experiment and hot-pressed sample are part of a study recently published by Précigout and Stünitz^25^. To produce the starting material, we crushed gem-quality olivine (Fo_91_) and diopside (Wo_51_En_48_Fs_1_) in an agate mortar, and sorted the grains according to their size using a decantation method with distilled water. For olivine, we used a grain size ranging from 1 to 10 µm with a mean of 2 µm. For clinopyroxene (CPx), we used coarser CPx grains between 40 and 125 µm. Olivine and pyroxene powders were mixed at a ratio of 70 to 30 wt% in a solution of ethanol following the procedure described in de Ronde *et al*.^36^. The powder was then placed between two alumina shear pistons cut at 45° to the piston axis, wrapped in a nickel foil to apply Ni/NiO buffering and enclosed in a platinum jacket welded at both ends. The shear piston surfaces have been previously roughened using 120-grit corundum paper. Before welding, 0.2 wt.% of distilled water was added. These amounts exceed the solubility of water in olivine and pyroxene at our experimental conditions^37,38^. Such wet conditions and grain size were chosen to ensure that (1) olivine deformed in the diffusion creep regime^39–41^, and (2) initial grains were large enough to then observe significant grain size reduction, if applicable. Few grains of orthopyroxene (enstatite; ~0.2 wt%) and amphibole (tremolite; ~0.8 wt%) were present in the CPx powder despite initial handpicking.

### Experimental set-up

Both the deformation and hot-pressing were conducted in a solid medium (NaCl) Griggs-type apparatus^42,43^ at Tromsø University (Norway). Temperature and pressure were alternately increased over several hours up to the chosen experimental conditions (900 °C and 1.2 GPa), which were applied for 24 h before deformation to hot-press the sample *in situ*. The deformation piston (σ_1_-piston) was subsequently advanced at a constant rate, first through the top lead piece, and then touching the alumina piston (see Supplementary Fig. S1a), so that the sample started to deform several hours after the σ_1_-piston began to advance. At the end of the experiment, the sample was quenched from 900 °C to 200 °C in 2 minutes, giving rise to a rapid decrease of pressure and differential stress until the temperature stabilised at 200 °C. The sample was then decompressed at a rate of ~5 MPa per minute, keeping the differential stress ~100 MPa above the confining pressure to reduce the formation of unloading cracks. Temperature was finally dropped to 30 °C when the confining pressure was lower than 100 MPa. After the experiment, the sample was impregnated with epoxy, and then cut along the piston axis for thin sections. To produce the hot-pressed, reference sample, we followed the same procedure, but without deforming the sample over the same duration of experiment (67 h).

### Secondary ion mass spectrometry (SIMS)

SIMS measurements are available in Supplementary Table S1. They have been performed on gold-coated (10 nm thickness) thin sections using the Cameca IMS 1280 HR at the CRPG (Nancy, France). 24 h before analysis, the thin sections were stored under vacuum in the sample storage chamber of the SIMS at a pressure of ~10^−8^ torr. A liquid nitrogen cold trap and a sublimation pump were used to reduce the H_2_O background and keep the vacuum below 3 × 10^−9^ torr in the sample chamber (except for very few spots (11) that were between 3 and 6 × 10^−9^ torr; see Supplementary Table S1). Samples were sputtered with a 2-3 nA, 10 kV primary beam of ^133^Cs^+^ focused to a ~10 μm spot. A raster of 10 × 10 µm was used during the 90 seconds of presputtering and of 5 × 5 µm during the analysis. The field aperture was closed to 1,000 µm to eliminate any secondary ion signal from the spot margin. The energy slit closed to 30 eV was manually shifted by ~4,000 digits (corresponding to an offset of ~40 eV) in order to keep ~5% of the initial signal. By doing this, we remove secondary ions with low energy and thus we eliminated potential hydrid contamination. Since variations of secondary ionization yields due to matrix effects preferentially affect low energy ions, this setting allows to keep it negligible compared to the analytical uncertainties. A mass-resolving power of ~7000, enough to resolve ^17^O from ^16^OH and ^30^Si from ^29^SiH was applied. ^16^O^−^ (3 s counting time), ^17^O^−^ (4 s), ^16^OH^−^ (12 s), ^28^Si^−^ (3 s) and ^30^Si^−^ (3 s) ions were counted on the axial electron multiplier or Faraday cup during 10 cycles. A set of reference glasses (ALV1833-11, MC-84, NWcoulée, Panum dome and PMR 53) and pyrope (MON 9) with SiO_2_ contents varying from 42.2 to 76.45% and H_2_O contents varying from 56 ppm to 1.17% were used to create the calibration line, which we used to calibrate H_2_O contents of unknown samples. Using the calibration line, the accuracy of SIMS analyses (average relative deviation of the measured value compared to the true value) was estimated to be 5% relative. The background concentration of H_2_O estimated from the calibration line is ~60 ppm. For data interpolation, we then used the MATLAB software with the *griddata* function (*natural* option).

### Electron backscatter diffraction (EBSD)

EBSD was employed for mapping polished sample surfaces (diamond paste of 0.25 μm followed by colloidal silica) with an EDAX Pegasus system at 20 kV and using a working distance of 15 mm. All maps were acquired using a step size between 0.1 and 0.2 μm over areas that averaged ~100 × 100 μm^2^ (see Supplementary Fig. S5). The data from EBSD maps were processed and “cleaned” using OIM - EDAX software. The “clean-up” procedure involved two iterations of grain dilation and the standardization of both the grain fit and confidence index. The grain size and interface density were then calculated using the open-source MTEX toolbox^44,45^ for MATLAB. To do so, we considered equivalent circular diameters of grains having a minimum of 5 indexed pixels adjacent to each other, as well as a minimum misorientation angle of 10° to define grain boundaries.

## Supplementary information

Supplementary information
