# Supplementary material for: Excess water storage induced by viscous strain localization during high-pressure shear experiment
Source: Sci Rep. 2019 Mar 5;9:3463. doi: 10.1038/s41598-019-40020-y (PMC6401144; doi:10.1038/s41598-019-40020-y)
Supplement: Supplementary file 1 — Supplementary information [file 41598_2019_40020_MOESM1_ESM.pdf]

Supplementary information for

Excess water storage induced by viscous strain localization during high-pressure shear experiment

Jacques Précigout*, Holger Stünitz and Johan Villeneuve

*To whom correspondence should be addressed: jacques.precigout@univ-orleans.fr

This pdf file includes:

- Figure S1: Sample assembly, mechanical output and hot-pressing sample.
- Figure S2: Sample used to characterize the depth of SIMS analyses
- Figure S3: Distribution of macro-porosity across the shear zone
- Figure S4: EBSD maps and local areas used to characterize the interface density across the hot-pressed sample.
- Figure S5: EBSD maps and local areas used to characterize the interface density across the strain gradient of the deformed sample.
- Table S1: SIMS datasheet
- Table S2: Datasheet for grain size calculation

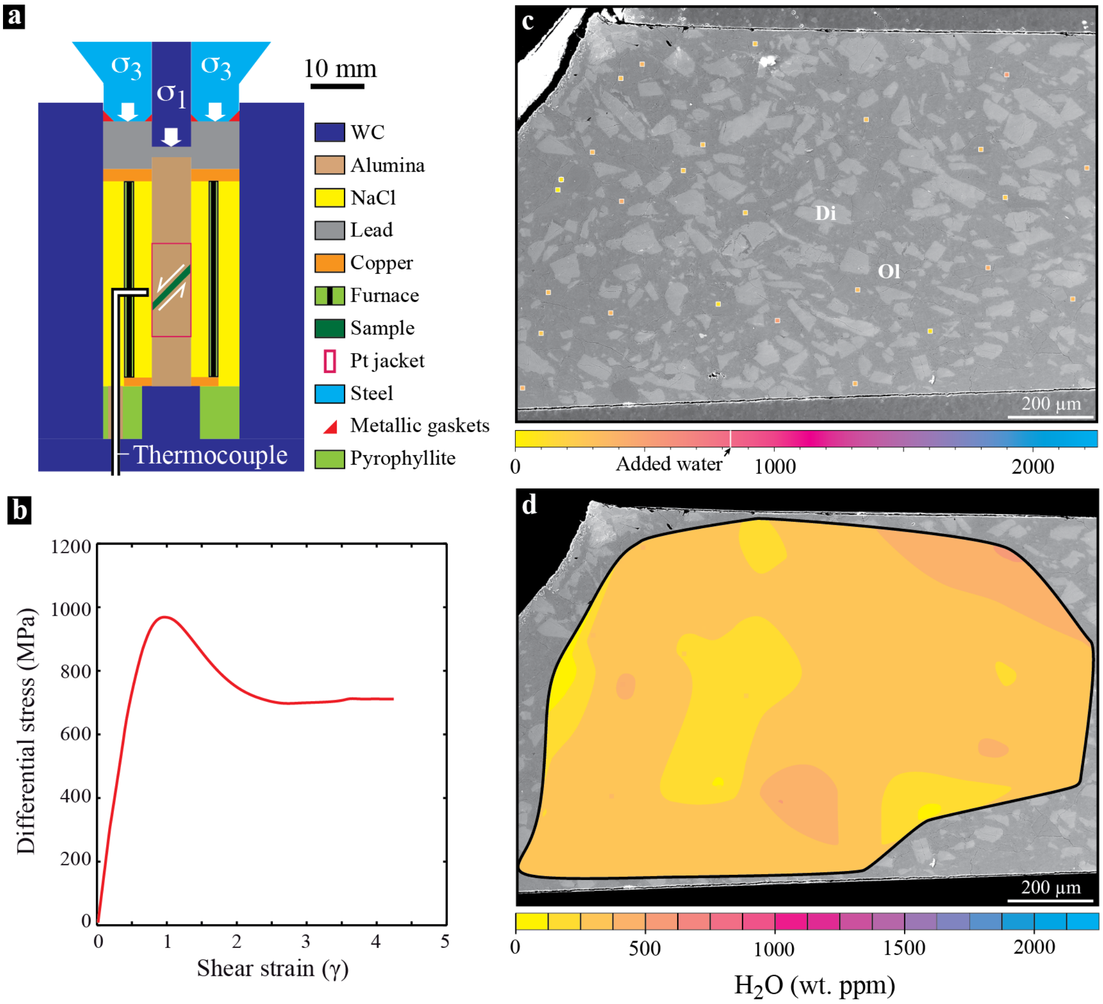

Figure S1 | Sample assembly, mechanical output and hot-pressing sample. a) Non-coaxial sample assembly used to deform the olivine (Ol) + diopside (Di) powder at 900 °C, 1.2 GPa and 2.10^{-5} s^{-1} in the Tullis-modified Griggs-type apparatus[1]. σ_1 and σ_3 are the principal stresses applied on the sample by top pistons. **b)** Differential stress versus shear gamma (γ) strain recorded during the deformation experiment. For further details, the readers are referred to Précigout and Stünitz[1]. **c)** Backscattered electron (BSE) image of a hot-pressed sample maintained under the same conditions as the deformed sample in terms of temperature (900 °C), pressure (1.2 GPa) and duration (67 h). Coloured squares show the location of the SIMS spots used to characterize the distribution of H_2O across the sample. Each colour refers to the amount of H_2O. **d)** Data interpolation of the SIMS dataset using the *griddata* function of MATLAB. The H_2O content is shown with colour coding every increment of 125 ppm.

[1] Précigout, J. & Stünitz, H. Evidence of phase nucleation during olivine diffusion creep: A new perspective for mantle strain localisation. *Earth. Planet. Sci. Lett.* **455**, 94-105 (2016).

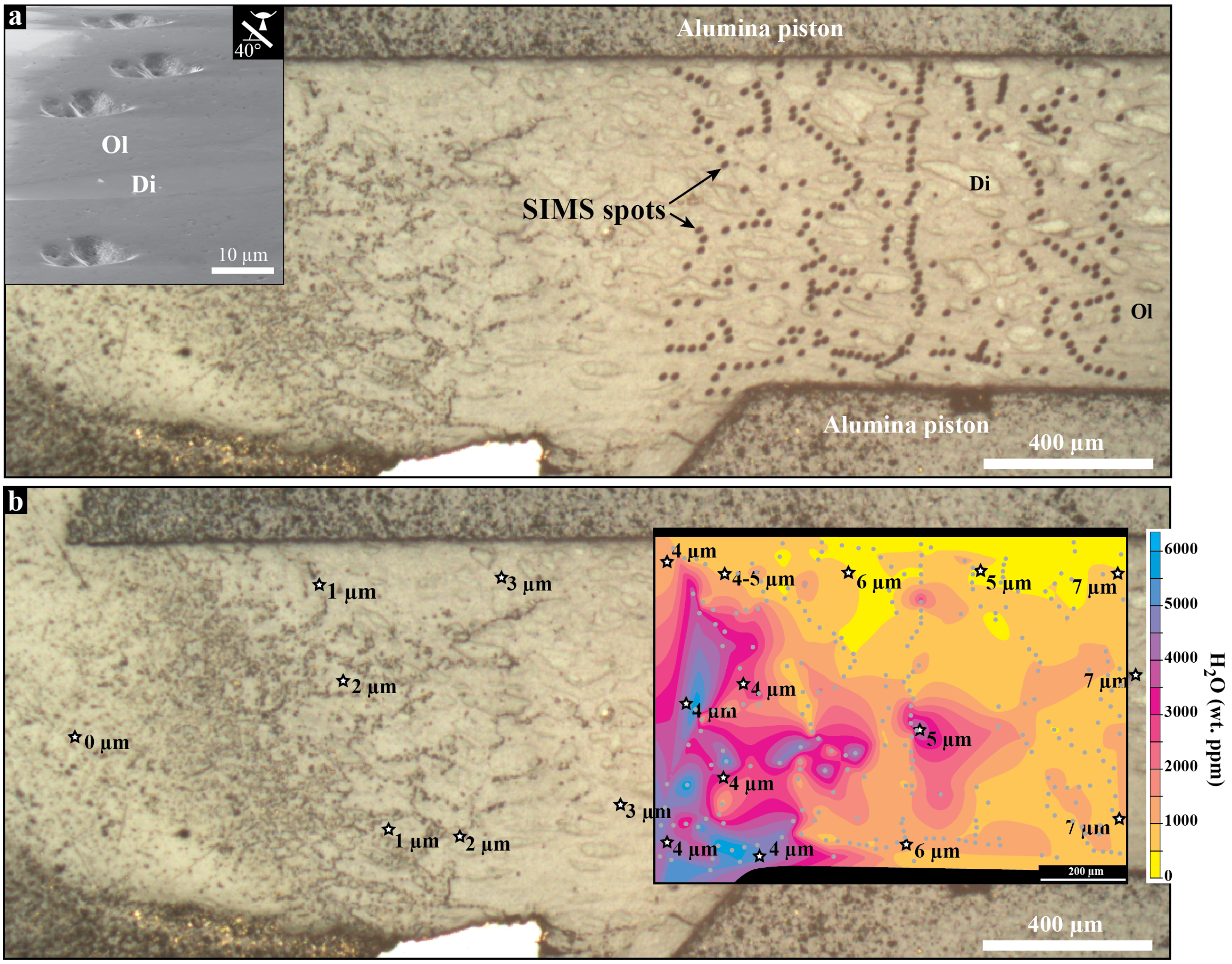

Figure S2 | Estimated depth of SIMS analyses. a) Thin section under reflected light of the deformed sample containing 0.2 wt% of added water (1330 ppm). The area is the same as the sample shown in the paper (same sample, but different thin section). The top left inset shows a BSE image that gives a better view of the SIMS spots on the sample surface inclined at 40°. **b)** Data mapping of the SIMS dataset (*griddata* function on MATLAB with increment every 250 ppm H_2O), which highlights very high H_2O content on the left. This concentration is an artefact due to the presence of epoxy below the sample, giving the opportunity to estimate the depth of a SIMS analysis (grey dots) in our analytical conditions. As shown by local measurements (stars) of sample thickness across the sample area, we estimate a depth of around 4 µm. Ol = olivine matrix; Di = Diopside.

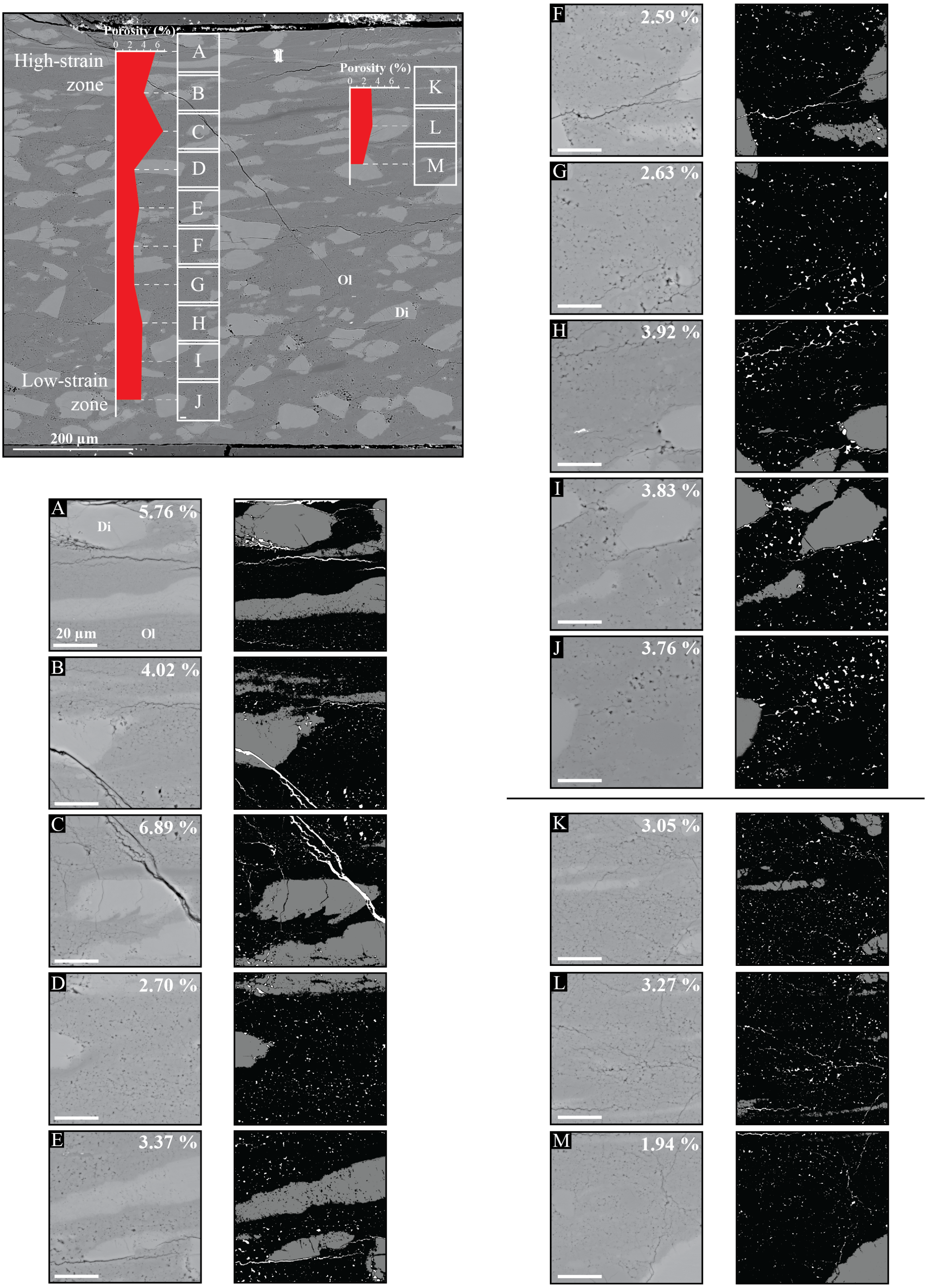

Figure S3 | Distribution of the macro-porosity across the shear zone. From the low- to high-strain zones, the macro-porosity is highlighted from BSE images (from **A** to **M**) using phase contrast between pores (black) and crystals (grey). The maps are located on the BSE image in the top left corner. The percentage of macro-pores is estimated for each map treated with MATLAB to isolate pixels of olivine (black) from diopside (grey) and pores, including cracks (white). The percentage only considers the amount of white pixels (pores) with respect to black pixels (olivine). While the H_2O content increases from the low- to high-strain zone (see Fig. 1 of the manuscript), the macro-porosity does not change significantly and remains around 3-4 % (see cross-sections on the top left image). Ol = olivine; Di = Diopside.

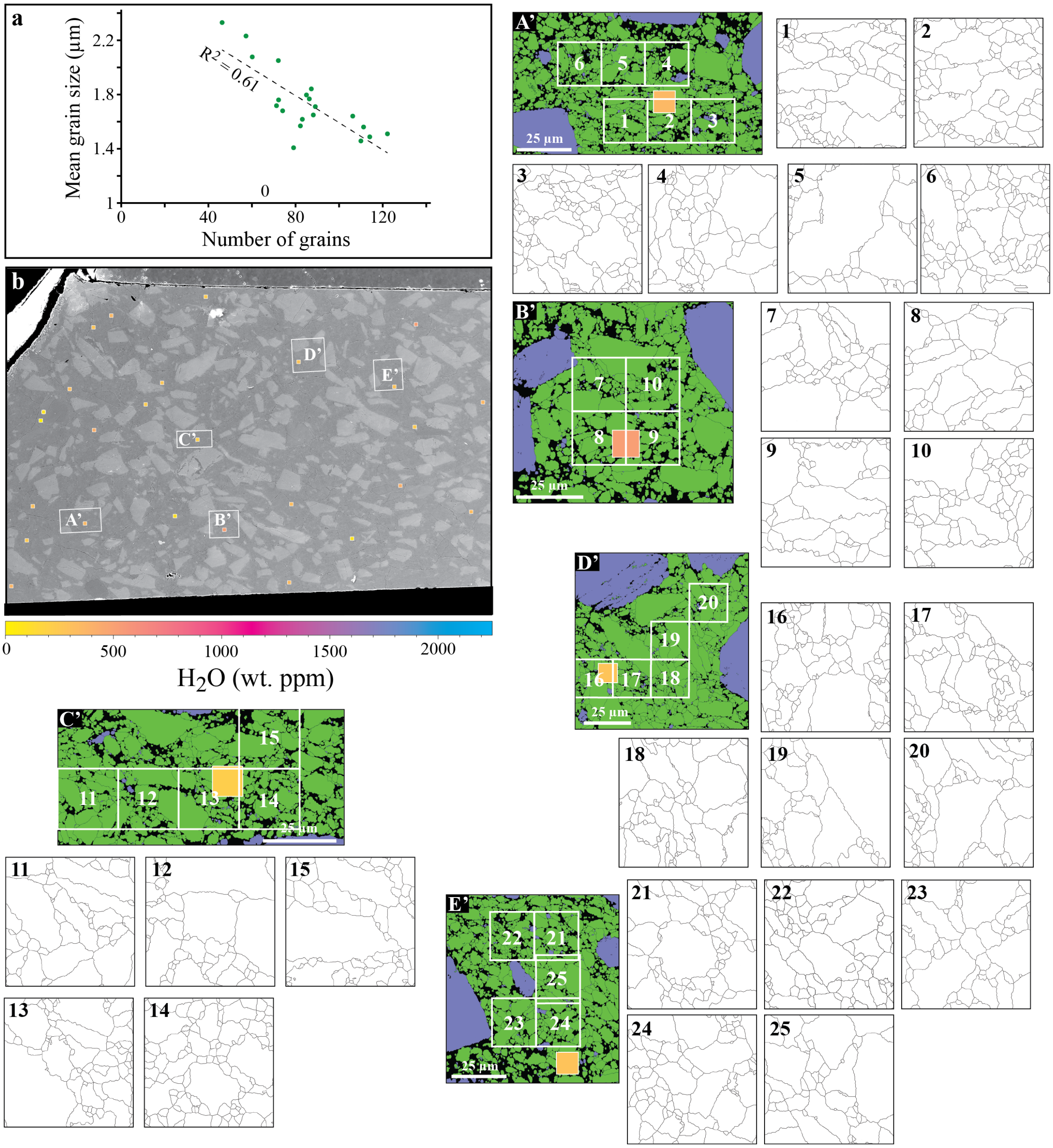

Figure S4 | Grain size and interface density across the hot-pressed sample. **a)** Mean grain size vs. number of grains calculated over areas of 20×20 µm^2 within 5 EBSD maps (shown and located in Supplementary Fig. S4b). Despite an important variation of grain size between 1.4 and 2.4 µm, the good correlation ($R^2 = 0.61$) with the number of grains confirms that the EBSD maps give a correct estimate of grain size. **b)** EBSD maps and grain boundary maps used to calculate the interface densities of the olivine matrix across the hot-pressed sample. From **A'** to **E'**, the interface densities have been calculated over areas of 20×20 µm^2 located on the EBSD maps. The maps are also located on the BSE image, which contains the SIMS spots and measured H_2O content used to estimate the loss of H_2O occurring during an experiment.

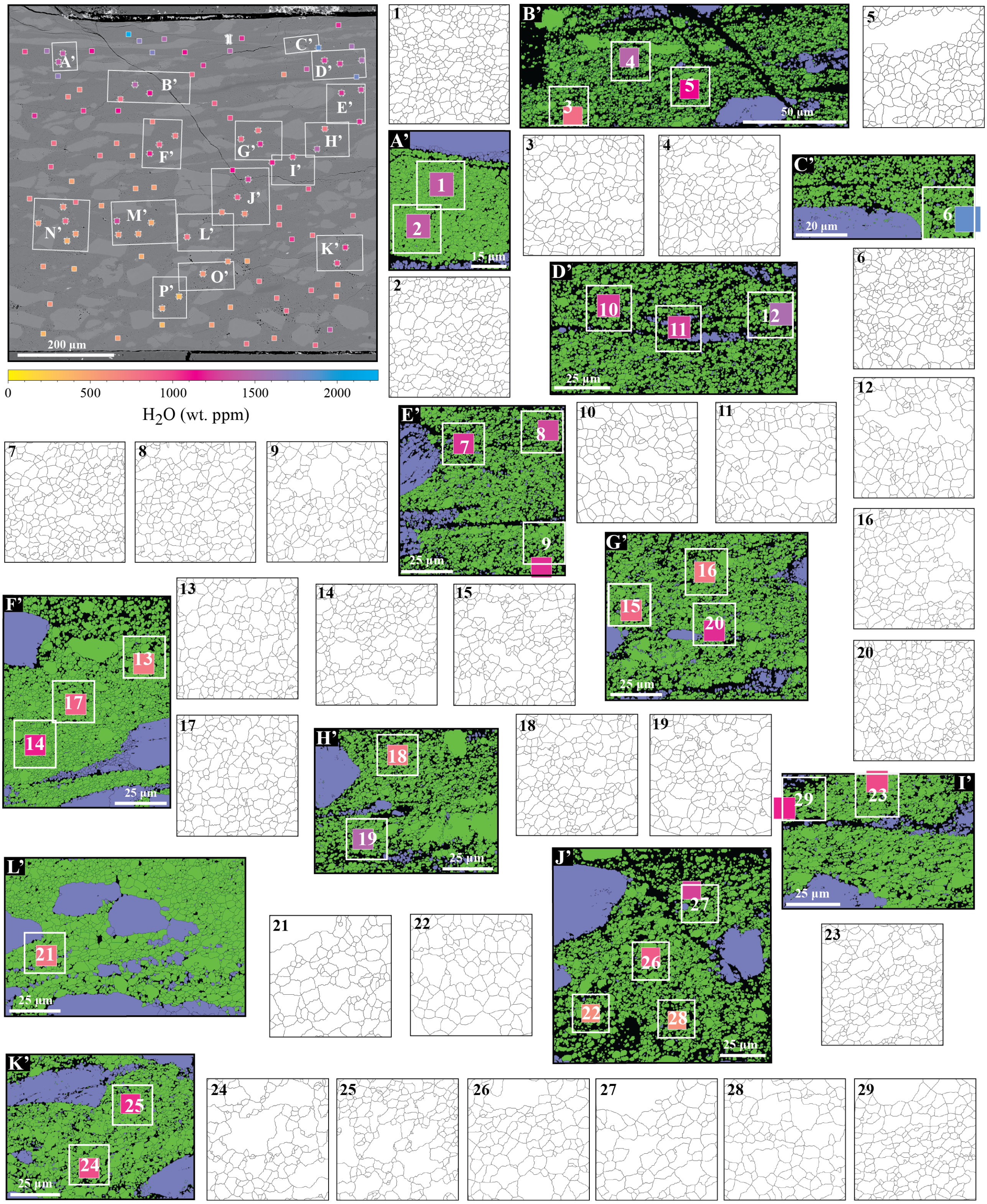

Figure S5 | Interface density across the strain gradient of the deformed sample. From **A'** to **P'**, we show the EBSD and grain boundary maps used to calculate the interface densities around each SIMS spot. Each map is located on the BSE image (top left). The grain boundaries are shown on 20×20 µm^2 maps and located on their respective EBSD map with the position of the related SIMS spot and amount of H_2O (colour coding). The olivine, diopside and pores, including non-indexed points, are shown in green, light blue and black, respectively. The pores are not shown on grain boundary maps.

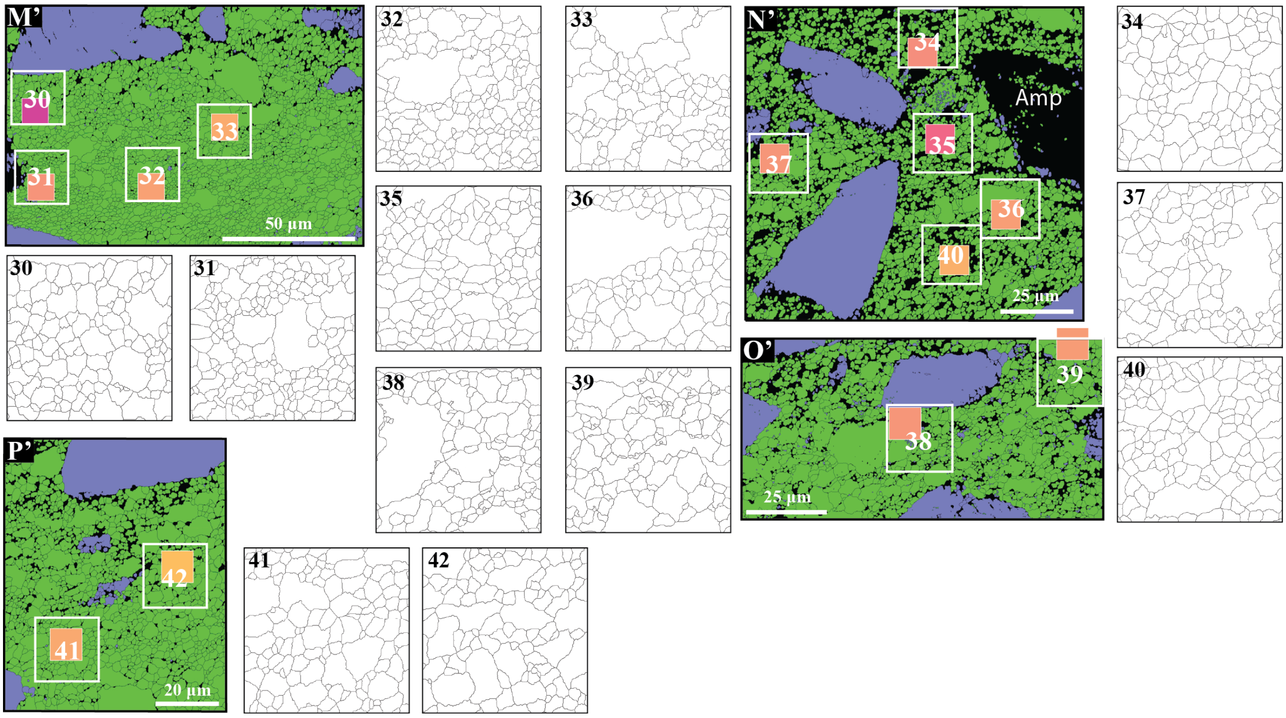

Figure S5 (continued)

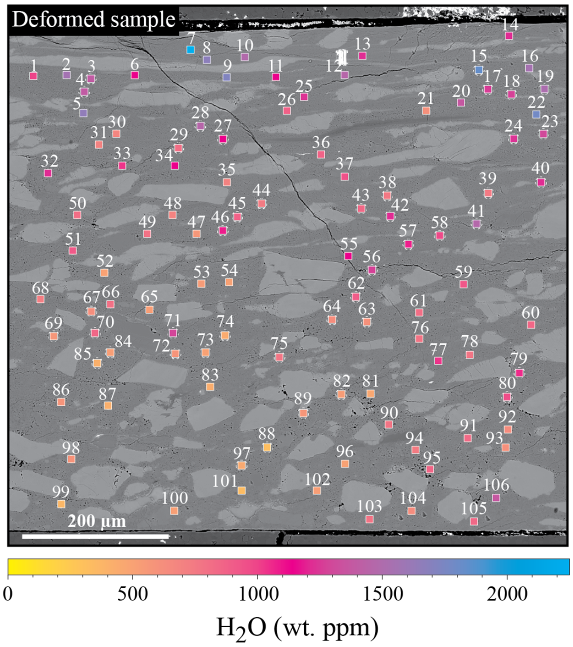

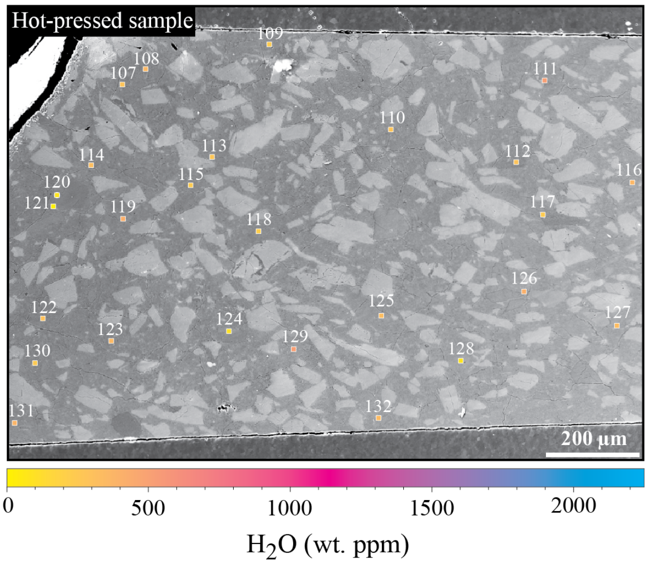

Analysis number	Chamber pressure	Primary intensity	^{16}O	$^{16}O^{1}H$	^{28}Si	^{30}Si	$^{28}Si/^{30}Si$	$^{16}O^{1}H/^{28}Si$	error	$^{16}O^{1}H/^{30}Si$	error	$^{1}H_2{}^{16}O/^{28}Si^{16}O_2$	H_2O (wt. ppm)	error
**1**	1.52E-09	2.12E-09	2.46E+07	5187.62	7.89E+05	2.52E+04	31.40	6.56E-03	1.65E-04	2.06E-01	5.11E-03	2.51E-03	**1002**	62
**2**	1.53E-09	2.10E-09	2.54E+07	8328.77	8.24E+05	2.63E+04	31.31	1.01E-02	1.83E-04	3.16E-01	5.51E-03	3.89E-03	**1556**	85
**3**	2.01E-09	1.92E-09	2.35E+07	7088.28	7.79E+05	2.51E+04	31.11	9.08E-03	1.97E-04	2.83E-01	6.11E-03	3.49E-03	**1397**	82
**4**	2.01E-09	1.89E-09	2.33E+07	6863.81	7.58E+05	2.42E+04	31.30	9.03E-03	2.51E-04	2.83E-01	7.81E-03	3.48E-03	**1390**	90
**5**	2.02E-09	1.87E-09	2.19E+07	7739.26	7.23E+05	2.32E+04	31.23	1.07E-02	5.76E-04	3.33E-01	1.79E-02	4.12E-03	**1648**	151
**6**	2.04E-09	1.94E-09	2.45E+07	6312.45	8.19E+05	2.63E+04	31.15	7.70E-03	6.88E-05	2.40E-01	2.27E-03	2.95E-03	**1180**	53
**7**	2.11E-09	1.93E-09	2.18E+07	10194.67	7.05E+05	2.26E+04	31.21	1.44E-02	7.54E-05	4.50E-01	2.68E-03	5.59E-03	**2235**	92
**8**	2.1E-09	1.95E-09	2.31E+07	8307.26	7.64E+05	2.46E+04	31.17	1.08E-02	8.43E-05	3.38E-01	2.79E-03	4.18E-03	**1673**	74
**9**	2.04E-09	1.93E-09	2.25E+07	8473.05	7.59E+05	2.43E+04	31.24	1.12E-02	1.24E-04	3.48E-01	3.69E-03	4.31E-03	**1723**	82
**10**	2.12E-09	1.95E-09	1.88E+07	5749.87	5.95E+05	1.90E+04	31.32	9.63E-03	6.84E-05	3.02E-01	2.15E-03	3.71E-03	**1484**	64
**11**	2.16E-09	1.96E-09	1.71E+07	4057.05	5.37E+05	1.72E+04	31.39	7.53E-03	7.95E-05	2.36E-01	2.55E-03	2.88E-03	**1154**	54
**12**	2.2E-09	2.03E-09	3.34E+07	11974.93	1.12E+06	3.63E+04	30.87	9.47E-03	2.69E-04	2.92E-01	8.69E-03	3.65E-03	**1459**	95
**13**	1.91E-09	1.88E-09	3.73E+07	9620.01	1.24E+06	3.98E+04	31.07	7.77E-03	8.07E-05	2.41E-01	2.52E-03	2.98E-03	**1192**	56
**14**	1.55E-09	2.14E-09	2.64E+07	6.94E+03	8.76E+05	2.81E+04	31.29	7.91E-03	1.04E-04	2.47E-01	2.93E-03	3.03E-03	**1213**	60
**15**	1.94E-09	2.13E-09	2.70E+07	10795.17	9.09E+05	2.92E+04	31.20	1.18E-02	3.56E-04	3.70E-01	1.18E-02	4.58E-03	**1832**	123
**16**	1.98E-09	2.16E-09	2.60E+07	8544.97	8.55E+05	2.75E+04	31.14	1.00E-02	5.51E-05	3.10E-01	2.26E-03	3.86E-03	**1545**	64
**17**	1.95E-09	2.14E-09	2.75E+07	7548.55	9.24E+05	2.97E+04	31.14	8.16E-03	5.18E-05	2.54E-01	1.63E-03	3.13E-03	**1253**	53
**18**	1.99E-09	2.17E-09	2.54E+07	7140.32	8.65E+05	2.79E+04	31.03	8.25E-03	1.35E-04	2.56E-01	4.19E-03	3.17E-03	**1267**	67
**19**	1.55E-09	2.11E-09	2.64E+07	8752.65	8.81E+05	2.82E+04	31.20	9.83E-03	1.00E-04	3.10E-01	3.88E-03	3.79E-03	**1515**	70
**20**	1.76E-09	2.13E-09	3.06E+07	9000.36	1.02E+06	3.27E+04	31.08	8.92E-03	6.14E-05	2.76E-01	8.34E-04	3.43E-03	**1372**	59
**21**	1.83E-09	2.09E-09	2.64E+07	3552.13	8.75E+05	2.82E+04	31.06	4.05E-03	1.48E-04	1.26E-01	4.55E-03	1.52E-03	**608**	46
**22**	2.04E-09	2.17E-09	2.66E+07	10103.32	8.77E+05	2.82E+04	31.13	1.15E-02	3.77E-04	3.58E-01	1.17E-02	4.45E-03	**1779**	124
**23**	2.05E-09	2.18E-09	2.72E+07	7619.57	8.98E+05	2.88E+04	31.20	8.46E-03	1.59E-04	2.64E-01	5.02E-03	3.25E-03	**1300**	72
**24**	2.15E-09	2.18E-09	2.78E+07	7389.11	9.18E+05	2.96E+04	31.08	8.03E-03	1.65E-04	2.50E-01	5.09E-03	3.08E-03	**1233**	71
**25**	1.57E-09	2.18E-09	6.99E+06	1818.94	2.25E+05	7.24E+03	31.21	8.04E-03	9.20E-05	2.49E-01	1.97E-03	3.09E-03	**1235**	59
**26**	2.25E-09	2.05E-09	2.28E+07	4378.05	7.38E+05	2.37E+04	31.20	5.76E-03	8.91E-05	1.80E-01	2.72E-03	2.19E-03	**876**	46
**27**	1.99E-09	1.85E-09	2.36E+07	5723.43	7.80E+05	2.51E+04	31.26	7.31E-03	1.33E-04	2.28E-01	4.16E-03	2.80E-03	**1120**	62
**28**	1.99E-09	1.86E-09	2.40E+07	7664.64	8.01E+05	2.58E+04	31.15	9.44E-03	1.25E-04	2.94E-01	3.70E-03	3.63E-03	**1454**	72
**29**	1.97E-09	1.84E-09	2.37E+07	4213.27	7.89E+05	2.53E+04	31.23	5.32E-03	5.93E-05	1.66E-01	1.87E-03	2.02E-03	**808**	39
**30**	1.95E-09	1.81E-09	2.21E+07	3259.42	7.25E+05	2.33E+04	31.16	4.48E-03	1.08E-04	1.39E-01	3.49E-03	1.69E-03	**675**	42
**31**	1.94E-09	1.81E-09	2.24E+07	3414.41	7.31E+05	2.35E+04	31.19	4.66E-03	1.17E-04	1.45E-01	3.66E-03	1.76E-03	**704**	44
**32**	1.95E-09	1.82E-09	2.20E+07	5636.19	7.22E+05	2.32E+04	31.19	7.78E-03	2.09E-04	2.43E-01	6.45E-03	2.98E-03	**1194**	76
**33**	1.96E-09	1.83E-09	2.24E+07	5152.29	7.61E+05	2.45E+04	31.09	6.75E-03	2.29E-04	2.10E-01	7.15E-03	2.58E-03	**1032**	74
**34**	1.93E-09	1.82E-09	2.25E+07	5606.98	7.47E+05	2.40E+04	31.09	7.64E-03	1.64E-04	2.38E-01	4.96E-03	2.93E-03	**1172**	68
**35**	1.94E-09	1.82E-09	2.22E+07	3469.32	7.33E+05	2.35E+04	31.22	4.72E-03	1.04E-04	1.47E-01	3.26E-03	1.78E-03	**713**	42
**36**	2.26E-09	2.01E-09	2.57E+07	4912.8	8.57E+05	2.76E+04	31.10	5.72E-03	6.21E-05	1.78E-01	1.68E-03	2.17E-03	**870**	41
**37**	2.29E-09	1.97E-09	2.60E+07	5571.73	8.77E+05	2.82E+04	31.11	6.23E-03	1.12E-04	1.94E-01	3.47E-03	2.38E-03	**950**	52
**38**	1.79E-09	2.09E-09	2.57E+07	4312.54	8.47E+05	2.72E+04	31.20	5.01E-03	4.96E-05	1.56E-01	1.57E-03	1.90E-03	**758**	35
**39**	2.23E-09	2.21E-09	2.70E+07	4391.74	8.86E+05	2.86E+04	30.97	4.95E-03	6.90E-05	1.53E-01	2.04E-03	1.87E-03	**750**	38
**40**	2.17E-09	2.19E-09	2.68E+07	7047.77	8.80E+05	2.83E+04	31.12	7.89E-03	1.25E-04	2.49E-01	5.05E-03	3.03E-03	**1211**	63
**41**	1.87E-09	2.10E-09	2.55E+07	8177.66	8.56E+05	2.76E+04	31.06	9.54E-03	2.46E-04	2.96E-01	7.64E-03	3.67E-03	**1470**	92
**42**	1.74E-09	2.09E-09	2.50E+07	6233.57	8.15E+05	2.61E+04	31.23	7.63E-03	1.80E-04	2.39E-01	5.58E-03	2.93E-03	**1170**	71
**43**	2.33E-09	2.00E-09	2.52E+07	4571.12	8.29E+05	2.67E+04	31.10	5.54E-03	4.79E-05	1.72E-01	1.56E-03	2.11E-03	**842**	38
**44**	1.67E-09	2.16E-09	2.68E+07	4385.18	8.95E+05	2.87E+04	31.14	4.89E-03	1.59E-04	1.52E-01	4.84E-03	1.85E-03	**741**	52

Table S1 | SIMS datasheet. While BSE images show the location of each SIMS analysis across the deformed and hot-pressed samples, the table gives the analytical conditions together with the oxygen, hydroxyl and silicon contents. The H_2O contents (in red) and related errors have been calculated from these recordings (see the methods section of the manuscript for further details).

Analysis number	Chamber pressure	Primary intensity	^{16}O	$^{16}O^{1}H$	^{28}Si	^{30}Si	$^{28}Si/^{30}Si$	$^{16}O^{1}H/^{28}Si$	error	$^{16}O^{1}H/^{30}Si$	error	$^{1}H_2{}^{16}O/^{28}Si^{16}O_2$	H_2O (wt ppm)	error
**45**	1.93E-09	1.81E-09	2.18E+07	4088.68	7.11E+05	2.28E+04	31.26	5.73E-03	9.65E-05	1.79E-01	3.07E-03	2.18E-03	872	47
**46**	1.9E-09	1.81E-09	2.31E+07	5345.27	7.56E+05	2.44E+04	31.08	7.06E-03	4.09E-04	2.20E-01	1.26E-02	2.70E-03	1081	104
**47**	1.88E-09	1.82E-09	2.24E+07	2761.18	7.33E+05	2.35E+04	31.24	3.75E-03	6.97E-05	1.17E-01	2.14E-03	1.40E-03	561	31
**48**	1.89E-09	1.82E-09	2.22E+07	3368.22	7.32E+05	2.35E+04	31.20	4.59E-03	1.23E-04	1.43E-01	3.91E-03	1.73E-03	692	45
**49**	1.85E-09	1.86E-09	2.22E+07	3793.31	7.26E+05	2.33E+04	31.33	5.21E-03	1.13E-04	1.63E-01	3.42E-03	1.97E-03	789	46
**50**	1.84E-09	1.90E-09	2.39E+07	4232.63	7.88E+05	2.53E+04	31.24	5.35E-03	1.81E-04	1.67E-01	5.77E-03	2.03E-03	813	58
**51**	1.79E-09	1.92E-09	2.31E+07	4586.54	7.59E+05	2.44E+04	31.15	6.03E-03	1.63E-04	1.88E-01	5.09E-03	2.30E-03	919	59
**52**	1.78E-09	1.92E-09	2.27E+07	2467.65	7.40E+05	2.38E+04	31.12	3.33E-03	9.24E-05	1.04E-01	2.78E-03	1.24E-03	495	33
**53**	1.71E-09	1.88E-09	2.18E+07	2559.9	7.03E+05	2.26E+04	31.13	3.59E-03	4.74E-05	1.13E-01	1.90E-03	1.34E-03	536	27
**54**	1.68E-09	1.85E-09	2.34E+07	2602.76	7.71E+05	2.48E+04	31.23	3.36E-03	5.69E-05	1.04E-01	1.38E-03	1.25E-03	500	27
**55**	2.39E-09	2.00E-09	2.38E+07	5729.36	7.76E+05	2.49E+04	31.16	7.37E-03	3.09E-04	2.30E-01	9.60E-03	2.82E-03	1129	90
**56**	2.41E-09	1.98E-09	2.38E+07	6351.27	7.67E+05	2.46E+04	31.21	8.27E-03	1.18E-04	2.58E-01	3.54E-03	3.18E-03	1270	64
**57**	2.26E-09	2.07E-09	2.51E+07	5936.78	8.32E+05	2.68E+04	31.12	7.13E-03	8.08E-04	2.22E-01	2.53E-02	2.73E-03	1091	168
**58**	1.54E-09	2.10E-09	2.60E+07	5473.43	8.50E+05	2.73E+04	31.19	6.42E-03	8.85E-05	2.00E-01	2.72E-03	2.45E-03	980	49
**59**	6.04E-09	2.06E-09	2.60E+07	5329.4	8.59E+05	2.76E+04	31.17	6.07E-03	9.24E-05	1.89E-01	2.76E-03	2.31E-03	924	48
**60**	2.3E-09	2.20E-09	2.71E+07	4901.53	8.77E+05	2.82E+04	31.09	5.58E-03	7.61E-05	1.73E-01	2.32E-03	2.12E-03	848	43
**61**	4.5E-09	2.05E-09	2.61E+07	4620.85	8.62E+05	2.77E+04	31.12	5.35E-03	1.14E-04	1.66E-01	3.54E-03	2.03E-03	812	47
**62**	2.45E-09	1.98E-09	2.48E+07	4905.57	8.15E+05	2.61E+04	31.25	6.00E-03	1.94E-04	1.88E-01	6.06E-03	2.29E-03	915	64
**63**	1.6E-09	2.20E-09	2.70E+07	3520.37	8.76E+05	2.81E+04	31.25	4.01E-03	7.34E-05	1.25E-01	2.32E-03	1.50E-03	602	33
**64**	2.5E-09	1.99E-09	2.46E+07	3401.09	7.96E+05	2.56E+04	31.10	4.17E-03	5.22E-05	1.30E-01	1.65E-03	1.57E-03	626	31
**65**	1.5E-09	2.14E-09	2.65E+07	3132.25	8.63E+05	2.76E+04	31.29	3.49E-03	1.01E-04	1.09E-01	3.16E-03	1.30E-03	521	35
**66**	1.75E-09	1.90E-09	2.26E+07	3854.79	7.32E+05	2.35E+04	31.22	5.25E-03	1.42E-04	1.64E-01	4.50E-03	1.99E-03	796	51
**67**	1.57E-09	1.72E-09	2.11E+07	2894.65	6.83E+05	2.19E+04	31.26	4.13E-03	8.47E-05	1.29E-01	2.69E-03	1.55E-03	621	36
**68**	1.59E-09	1.73E-09	2.14E+07	3216.57	6.89E+05	2.21E+04	31.33	4.65E-03	9.00E-05	1.46E-01	2.80E-03	1.76E-03	702	40
**69**	1.57E-09	1.72E-09	2.07E+07	2583.19	6.83E+05	2.19E+04	31.13	3.78E-03	5.32E-05	1.18E-01	1.69E-03	1.41E-03	565	29
**70**	1.54E-09	1.72E-09	2.03E+07	3682.62	6.50E+05	2.08E+04	31.33	5.58E-03	5.07E-05	1.75E-01	1.57E-03	2.12E-03	848	39
**71**	1.65E-09	1.82E-09	2.11E+07	5678.37	6.79E+05	2.18E+04	31.24	8.34E-03	1.11E-04	2.61E-01	3.45E-03	3.20E-03	1282	64
**72**	1.6E-09	1.77E-09	2.12E+07	2665.4	6.83E+05	2.19E+04	31.25	3.89E-03	8.24E-05	1.22E-01	2.65E-03	1.46E-03	583	34
**73**	1.61E-09	1.77E-09	2.11E+07	2296.52	6.76E+05	2.16E+04	31.36	3.38E-03	1.05E-04	1.06E-01	3.29E-03	1.26E-03	503	35
**74**	1.65E-09	1.80E-09	2.21E+07	2116.75	7.16E+05	2.30E+04	31.22	2.95E-03	5.62E-05	9.21E-02	1.79E-03	1.09E-03	435	25
**75**	2.56E-09	2.00E-09	2.40E+07	3991.08	7.80E+05	2.51E+04	31.14	5.12E-03	3.03E-04	1.59E-01	9.29E-03	1.94E-03	775	76
**76**	1.61E-09	2.20E-09	2.70E+07	4626.82	8.83E+05	2.82E+04	31.30	5.15E-03	7.25E-05	1.61E-01	2.39E-03	1.95E-03	781	40
**77**	3.93E-09	2.04E-09	2.52E+07	5571.17	8.24E+05	2.64E+04	31.22	6.75E-03	2.74E-04	2.11E-01	8.32E-03	2.58E-03	1032	81
**78**	2.38E-09	2.12E-09	2.72E+07	4556.51	8.95E+05	2.90E+04	30.95	5.08E-03	7.91E-05	1.57E-01	2.35E-03	1.92E-03	770	40
**79**	2.43E-09	2.06E-09	2.50E+07	5633.62	8.04E+05	2.60E+04	31.02	6.91E-03	8.11E-05	2.16E-01	2.91E-03	2.64E-03	1057	51
**80**	2.48E-09	2.05E-09	2.49E+07	5216.2	8.09E+05	2.64E+04	30.69	6.33E-03	6.11E-05	1.94E-01	2.13E-03	2.41E-03	966	45
**81**	3.37E-09	2.03E-09	2.52E+07	2600.2	8.27E+05	2.65E+04	31.21	3.14E-03	5.12E-05	9.79E-02	1.60E-03	1.16E-03	465	25
**82**	2.58E-09	2.01E-09	2.40E+07	2887.28	8.06E+05	2.59E+04	31.16	3.57E-03	6.52E-05	1.11E-01	2.11E-03	1.33E-03	533	30
**83**	1.6E-09	1.75E-09	2.06E+07	2202.6	6.56E+05	2.11E+04	31.15	2.90E-03	1.26E-04	9.05E-02	4.00E-03	1.07E-03	427	35
**84**	1.55E-09	1.72E-09	2.02E+07	2324.09	6.50E+05	2.09E+04	31.25	3.49E-03	6.70E-05	1.09E-01	2.12E-03	1.30E-03	521	29
**85**	1.53E-09	1.71E-09	1.97E+07	1773.44	6.33E+05	2.03E+04	31.18	2.79E-03	4.74E-05	8.71E-02	1.50E-03	1.03E-03	411	22
**86**	1.54E-09	1.70E-09	1.94E+07	2421.49	6.22E+05	2.00E+04	31.22	3.88E-03	1.27E-04	1.18E-01	3.17E-03	1.45E-03	581	41
**87**	1.53E-09	1.69E-09	1.98E+07	1818.78	6.33E+05	2.03E+04	31.23	2.84E-03	2.66E-05	8.86E-02	7.46E-04	1.04E-03	418	19
**88**	2.73E-09	2.02E-09	2.45E+07	1838.74	7.98E+05	2.56E+04	31.22	2.22E-03	7.30E-05	6.93E-02	2.26E-03	8.01E-04	321	23
**89**	2.63E-09	2.01E-09	2.44E+07	3040.47	7.98E+05	2.56E+04	31.16	3.80E-03	1.82E-04	1.18E-01	5.62E-03	1.42E-03	569	49
**90**	3.19E-09	2.02E-09	2.48E+07	4111.99	8.12E+05	2.61E+04	31.10	5.11E-03	6.02E-05	1.59E-01	1.82E-03	1.94E-03	774	37
**91**	2.76E-09	2.07E-09	2.50E+07	4443.9	8.02E+05	2.59E+04	31.01	5.47E-03	5.45E-05	1.70E-01	1.79E-03	2.08E-03	831	39
**92**	2.53E-09	2.04E-09	2.45E+07	3361.91	7.83E+05	2.53E+04	30.97	4.21E-03	3.88E-05	1.30E-01	1.27E-03	1.58E-03	633	29
**93**	2.65E-09	2.06E-09	2.52E+07	3251.5	8.02E+05	2.61E+04	30.76	4.05E-03	1.09E-04	1.25E-01	3.34E-03	1.52E-03	608	39
**94**	2.89E-09	2.07E-09	2.57E+07	4388.18	8.22E+05	2.66E+04	31.01	4.87E-03	2.83E-04	1.51E-01	8.84E-03	1.84E-03	736	72
**95**	3.05E-09	2.07E-09	2.49E+07	4321.25	8.06E+05	2.60E+04	31.05	5.35E-03	1.22E-04	1.66E-01	3.80E-03	2.03E-03	812	49
**96**	3.09E-09	2.02E-09	2.35E+07	2562.33	7.63E+05	2.45E+04	31.19	3.35E-03	5.02E-05	1.04E-01	1.63E-03	1.24E-03	498	26
**97**	2.81E-09	2.03E-09	2.34E+07	2325.79	7.53E+05	2.41E+04	31.29	3.08E-03	1.09E-04	9.65E-02	3.40E-03	1.14E-03	456	34
**98**	1.67E-09	1.68E-09	1.94E+07	2662.92	6.14E+05	1.96E+04	31.37	3.93E-03	1.26E-04	1.23E-01	3.99E-03	1.47E-03	589	41
**99**	1.96E-09	1.68E-09	1.79E+07	1415.55	5.65E+05	1.81E+04	31.31	2.50E-03	5.64E-05	7.82E-02	1.76E-03	9.11E-04	364	22
**100**	2.02E-09	1.68E-09	1.85E+07	2086.37	5.85E+05	1.87E+04	31.36	3.45E-03	7.12E-05	1.08E-01	2.15E-03	1.28E-03	513	30
**101**	2.84E-09	2.03E-09	2.31E+07	1792.89	7.43E+05	2.38E+04	31.21	2.34E-03	2.88E-05	7.30E-02	8.64E-04	8.50E-04	340	17
**102**	2.98E-09	2.02E-09	2.42E+07	2888.65	7.89E+05	2.54E+04	31.10	3.49E-03	1.50E-04	1.08E-01	4.68E-03	1.30E-03	520	43
**103**	3.65E-09	2.07E-09	2.32E+07	3745.88	7.23E+05	2.37E+04	30.45	5.17E-03	8.20E-05	1.57E-01	2.54E-03	1.96E-03	783	41
**104**	3.3E-09	2.07E-09	2.29E+07	3034.82	7.15E+05	2.31E+04	31.04	4.23E-03	2.54E-05	1.31E-01	8.18E-04	1.59E-03	636	27
**105**	6.85E-09	2.06E-09	2.32E+07	4110.39	7.33E+05	2.39E+04	30.73	5.53E-03	6.16E-05	1.70E-01	1.90E-03	2.10E-03	840	40
**106**	4.42E-09	2.05E-09	2.42E+07	6780.02	7.64E+05	2.49E+04	30.78	8.86E-03	1.91E-04	2.72E-01	5.69E-03	3.41E-03	1362	79
**107**	1.36E-09	2.05E-09	3.05E+07	2270.66	1.03E+06	3.36E+04	30.66	1.93E-03	2.75E-05	5.94E-02	8.43E-04	6.90E-04	276	14
**108**	1.44E-09	2.12E-09	3.04E+07	2391.53	1.02E+06	3.32E+04	30.73	2.34E-03	1.07E-04	7.20E-02	3.22E-03	8.50E-04	340	29
**109**	1.36E-09	2.04E-09	2.95E+07	1676.28	9.99E+05	3.27E+04	30.68	1.67E-03	5.76E-05	5.12E-02	1.66E-03	5.88E-04	235	18
**110**	1.42E-09	2.12E-09	3.06E+07	2083.14	1.04E+06	3.41E+04	30.48	1.93E-03	6.30E-05	5.88E-02	1.93E-03	6.88E-04	275	20
**111**	1.43E-09	2.10E-09	2.97E+07	3436.28	9.94E+05	3.24E+04	30.67	3.44E-03	7.96E-04	1.05E-01	2.42E-02	1.28E-03	512	145
**112**	1.41E-09	2.14E-09	3.08E+07	2149.91	1.04E+06	3.38E+04	30.69	1.97E-03	5.34E-05	6.04E-02	1.59E-03	7.04E-04	282	19
**113**	1.46E-09	2.13E-09	3.09E+07	2013.16	1.04E+06	3.40E+04	30.51	1.85E-03	8.75E-05	5.66E-02	2.71E-03	6.58E-04	263	23
**114**	2.97E-09	2.03E-09	2.97E+07	2322.5	1.01E+06	3.28E+04	30.79	2.19E-03	1.09E-04	6.74E-02	3.40E-03	7.90E-04	316	29

Table S1 (continued)

**Analysis number**	**Chamber pressure**	**Primary intensity**	**^{16}O**	**$^{16}O^1H$**	**^{28}Si**	**^{30}Si**	**$^{28}Si/^{30}Si$**	**$^{16}O^1H/^{28}Si$**	**error**	**$^{16}O^1H/^{30}Si$**	**error**	**$^1H_2{}^{16}O/^{28}Si^{16}O_2$**	**H_2O (wt ppm)**	**error**
**115**	1.48E-09	2.16E-09	3.13E+07	1747.49	1.05E+06	3.45E+04	30.48	1.64E-03	2.17E-05	5.01E-02	6.69E-04	5.75E-04	**230**	12
**116**	1.37E-09	2.05E-09	2.86E+07	2674.35	1.07E+06	3.49E+04	30.72	2.50E-03	9.53E-05	7.67E-02	2.94E-03	9.11E-04	**364**	28
**117**	1.37E-09	2.05E-09	2.81E+07	1757.53	1.07E+06	3.51E+04	30.65	1.64E-03	5.77E-05	5.03E-02	1.81E-03	5.74E-04	**230**	17
**118**	1.46E-09	2.17E-09	3.03E+07	1724.78	1.10E+06	3.64E+04	30.27	1.52E-03	2.76E-05	4.62E-02	8.05E-04	5.29E-04	**212**	12
**119**	1.53E-09	2.17E-09	3.12E+07	2949.41	1.03E+06	3.43E+04	30.08	2.79E-03	6.82E-05	8.41E-02	2.08E-03	1.03E-03	**411**	26
**120**	2.79E-09	2.01E-09	2.94E+07	499.12	9.82E+05	3.19E+04	30.81	5.08E-04	9.48E-06	1.56E-02	2.96E-04	1.30E-04	**52**	3
**121**	1.4E-09	2.11E-09	3.07E+07	199.49	1.02E+06	3.32E+04	30.72	1.96E-04	3.12E-06	6.02E-03	9.89E-05	7.86E-06	**3**	1
**122**	1.6E-09	2.19E-09	3.23E+07	2205.84	1.07E+06	3.54E+04	30.31	2.05E-03	6.23E-05	6.22E-02	1.88E-03	7.35E-04	**294**	21
**123**	1.66E-09	2.14E-09	3.14E+07	2489.85	1.02E+06	3.43E+04	29.82	2.43E-03	6.83E-05	7.25E-02	1.82E-03	8.85E-04	**354**	24
**124**	1.51E-09	2.19E-09	3.18E+07	903.87	1.06E+06	3.48E+04	30.67	8.35E-04	9.32E-06	2.56E-02	2.78E-04	2.59E-04	**104**	5
**125**	1.42E-09	2.13E-09	3.05E+07	2344.46	1.04E+06	3.39E+04	30.75	2.24E-03	6.23E-05	6.91E-02	1.93E-03	8.11E-04	**325**	22
**126**	1.41E-09	2.14E-09	3.04E+07	3419.18	1.02E+06	3.28E+04	30.34	2.81E-03	1.68E-04	8.90E-02	6.25E-03	1.03E-03	**413**	42
**127**	1.39E-09	2.07E-09	3.02E+07	2309.85	1.03E+06	3.36E+04	30.81	2.23E-03	3.39E-05	6.87E-02	1.08E-03	8.05E-04	**322**	17
**128**	1.38E-09	2.12E-09	3.17E+07	729.25	1.08E+06	3.51E+04	30.83	6.76E-04	1.18E-05	2.08E-02	3.69E-04	1.96E-04	**78**	5
**129**	1.52E-09	2.16E-09	3.09E+07	3670.57	1.06E+06	3.45E+04	30.59	3.42E-03	5.54E-05	1.05E-01	1.74E-03	1.27E-03	**509**	27
**130**	1.72E-09	2.09E-09	3.07E+07	1869.22	1.03E+06	3.37E+04	30.53	1.71E-03	9.22E-05	5.23E-02	2.84E-03	6.02E-04	**241**	23
**131**	1.53E-09	2.11E-09	3.12E+07	3037.73	1.02E+06	3.39E+04	30.08	2.57E-03	1.78E-04	7.72E-02	5.26E-03	9.40E-04	**376**	42
**132**	1.36E-09	2.04E-09	2.97E+07	2390.82	1.03E+06	3.38E+04	30.63	2.31E-03	8.80E-05	7.07E-02	2.64E-03	8.36E-04	**334**	26

Table S1 (continued)

Distance from α (µm)	Mean (µm)	Error (± µm)	Standard deviation (µm)	Number of grains
20	1.81	0.035233	0.59272	283
47	1.62	0.032364	0.54672	286
70	1.92	0.03864	0.58217	227
98	1.63	0.055869	0.79011	200
128	1.54	0.049181	0.73443	223
139	1.44	0.03584	0.59326	274
175	1.97	0.036547	0.56854	242
194	1.86	0.039082	0.52725	182
220	1.86	0.039873	0.62028	242
244	1.68	0.031716	0.57088	324
280	1.68	0.034674	0.50843	215
282	1.59	0.030976	0.51274	274
303	1.6	0.029629	0.50283	288
309	1.56	0.033396	0.50537	229
329	1.65	0.04109	0.58545	203
362	1.66	0.035284	0.52335	220
393	1.36	0.032516	0.58889	328
409	1.49	0.033577	0.61273	333
431	1.24	0.025067	0.5264	441
485	1.26	0.027308	0.5267	372
523	1.28	0.023744	0.4992	442
542	1.23	0.023801	0.4989	439
570	1.24	0.026346	0.53347	410
599	1.21	0.021657	0.49763	528
620	1.2	0.020587	0.46537	511
668	1.2	0.025414	0.5196	418
688	1.29	0.027465	0.54309	391
688	1.25	0.028163	0.5351	361
705	1.28	0.028496	0.53992	359
749	1.13	0.019377	0.44483	527

Table S2 | Datasheet for grain size calculation. As displayed in figure 2b of the manuscript, each value of mean grain size is given according to the distance of their respective area from the tip of the cross-section shown in figure 2a (α). For each area, the standard deviation of the log-normal distribution and number of grains have been used to calculate the error bar.
